# Ultrasound Detection of Intracranial Hypertension in Brain Injuries

**DOI:** 10.3389/fmed.2022.870808

**Published:** 2022-06-30

**Authors:** Livio Vitiello, Giulio Salerno, Maddalena De Bernardo, Olga D'Aniello, Luigi Capasso, Giuseppe Marotta, Nicola Rosa

**Affiliations:** ^1^Eye Unit, Department of Medicine, Surgery and Dentistry, “Scuola Medica Salernitana,” University of Salerno, Salerno, Italy; ^2^Corneal Transplant Unit, Azienda Sanitaria Locale Napoli 1, Naples, Italy; ^3^Eye Unit, Azienda Ospedaliera Universitaria “San Giovanni di Dio e Ruggi D'Aragona”, Salerno, Italy

**Keywords:** head trauma, intracranial pressure, optic nerve, optic nerve sheath diameter, ultrasonography

## Abstract

In recent years, the measurement of optic nerve sheath diameter with ultrasound to detect the presence of increased intracranial pressure has widely spread. It can be qualitatively and effectively used to identify intracranial hypertension. Intracranial pressure can rise due to acute injury, cerebral bleeding, hydrocephalus, brain tumors and other space-occupying abnormalities, and it is linked to a high death rate. The purpose of this review is to give a general overview of the most relevant scientific publications on ultrasonographic evaluation of the optic nerve in case of brain injuries published in the last 30 years, as well as to analyze the limits of the most extensively used B-scan approach. Fifty-two papers chosen from the PubMed medical database were analyzed in this review. Our findings revealed that ocular ultrasound is an useful diagnostic tool in the management of intracranial hypertension when it exceeds a certain value or after head trauma. As a result, an ultrasound of the optic nerve can be extremely helpful in guiding diagnosis and treatment. The blooming effect is one of the most critical restrictions to consider when using B-scan ultrasonography. Since amplitude-scan ultrasound, also known as A-scan, does not have this limit, these two diagnostic techniques should always be used together for a more full, accurate, and trustworthy ultrasound examination, ensuring more data objectivity.

## Introduction

Post-traumatic damage and other space-occupying lesions, such as cerebral hemorrhage, brain abscesses and subdural or epidural hematomas, can produce increased intracranial pressure (ICP) (1). Intracranial hypertension can also develop in the context of venous blood flow obstacles, such as hypoxia-ischemia and venous sinus thrombosis, increased cerebral volume, infections and inflammatory processes, and hepatic or hypertensive encephalopathy. Moreover, ICP is sometimes caused by an unknown cause (1).

Intraventricular catheterization and intraparenchymal probes are the gold standard procedures for measuring ICP, together with lumbar puncture. They entail various dangers, including infection and bleeding and are therefore contraindicated in individuals who require long-term monitoring or who are inclined to coagulopathy or platelet diseases (2).

Moreover, lumbar puncture is contraindicated in case of brain tumors (3).

Over the years, different non-invasive methods for evaluating ICP have been developed (4). Because of its feasibility, repeatability, safety, and lack of radiation hazard or known side effects, ultrasonographic measurement of optic nerve sheath diameter (ONSD) has come to be accepted (5).

ICP rise will result in increase in ONSD in the retrobulbar compartment because the sheath encircling the optic nerve is an outgrowth of the dura-mater and is filled with cerebrospinal fluid (CSF) (6). Ultrasound is a well-established tool for evaluating optic nerve sheath dilatation, which aids clinicians in the management of intracranial hypertension, when it exceeds a particular value (7, 8). Moreover, increased ICP following a head injury must be recognized quickly in order to receive effective treatment (1). Bedside ultrasonography, which is available in most trauma centers, should be practical and rapid to use in this clinical condition (9, 10), or could even be used in a prehospital setting (11).

Moreover, it has been shown to be effective in monitoring the changes of ICP in case of idiopathic intracranial hypertension (12). In addition, to detect the correlation between ONSD and ICP and to find a cut off value is mandatory to establish the role of US in detecting the presence of increased ICP.

Considering all the aforesaid reasons, the purpose of this paper is to review the effectiveness and reliability of ocular ultrasonography in monitoring ICP in case of traumatic brain injury (TBI) and space-occupying lesions, through the analysis of the published scientific literature in the last 30 years.

## Materials and Methods

We searched within the PubMed medical database and we entered search strings including terms related to “traumatic brain injury,” “ocular ultrasonography,” and “raised intracranial pressure.” The text terms were taken from current literature and/or associated bibliographies. Additional entries were found by manually searching bibliographies from the original searches.

The current study included only original researches, case reports, and case series on optic nerve ultrasonography examination for diagnosing intracranial hypertension and traumatic brain injury. The earliest date of publication was chosen for January 1990, and the search was completed in August 2021.

We found 125 items in our initial search, of which 13 were excluded because they are not directly related to the discussed topic. In addition, 15 papers dealing with increased ICP due to idiopathic intracranial hypertension and 45 papers related to surgical maneuvers were also excluded from the study.

Finally, 52 publications were included, 30 for intracranial hypertension due to space-occupying lesions and 22 for traumatic brain injury.

## Results

### ICP and Importance of the Measurement Methods

To get reproducible and reliable measurements of the ONSD is a very important topic. For such a purpose in the literature, several methods have been described.

The ONSD was measured for the first time by KC Ossoinig utilizing the so called Standardized A scan (7, 8).

Later on, several authors tried to measure the ONSD utilizing the B scan technique with transversal or coronal scans, as it is shown in Table 1.

**Table 1:** Potential cut-off to predict raised intracranial pressure.

**Study**	**No of patients**	**Methods of evaluation**	**ONSD cut-off point**	**Sensitivity**	**Specificity**
Kimberly et al. (13)	15	Linear ultrasoud probe (axial B-Scan)	5 mm	88% (95% CI: 47–99%)	93% (95% CI: 78–99%)
Adauayi et al. (14)	160 (80 controls)	Linear ultrasoud probe (axial B-Scan)	5.2 mm	81.2% (95% CI: 69.9–89.6%)	100% (95% CI: 71.5–100%)
Lee et al. (15)	134	Linear ultrasoud probe (transverse B-Scan)	5.5 mm (in Korean population)	99% (95% CI: 93–100%)	85% (95% CI: 66–95%)
Bolesch et al. (16)	45	Linear ultrasoud probe (transvere B-Scan)	5.7 mm	53.5%	100.0%
Salahuddin et al. (17)	102	Linear ultrasoud probe (transverse B-Scan)	5.7 mm (non-traumatic cerebral edema)	84% (95% CI: 70–87%)	71% (95% CI: 70–87%)
Wang et al. (18)	279	Linear ultrasoud probe (transverse and sagittal B-Scan)	4.1 mm (in Chinese population)	95%	92%
Komut et al. (19)	100	Linear ultrasoud probe (transverse and sagittal B-Scan)	4.7 mm	70%	86%
Moretti et al. (20)	63	Linear ultrasoud probe (transverse and sagittal B-Scan)	5.2 mm (in intracranial hemorrhages)	93% (95% CI: 77.2–99%)	74% (95% CI: 61.5–84%)
Moretti et al. (21)	53	Linear ultrasoud probe (transverse and sagittal B-Scan)	5.2 mm (in intracranial hemorrhages)	94% (95% CI: 88–100%)	76% (95% CI: 65–87%)
Major et al. (22)	26	Linear ultrasoud probe (B-Scan, with no specified plane)	Not detected	86% (95% CI: 42–99%)	100% (95% CI: 79–100%)

*ONSD, Optic nerve sheath diameter. 95% CI: 95% Confidence intervals*.

Most of the authors utilized the probe over the closed lids, measuring the distance between the borders of an hyporeflective area surrounding the optic nerve, 3 mm behind the optic nerve insertion. Not all the authors utilized this approach.

Chen et al. placed the B scan probe over the open eyes, on the corneal temporal limbus, after topical anesthesia (23).

Topcuoglu et al. (24) suggested to measure both the outermost hypoechogenic space, which they claimed to correspond to the subarachnoid space and the diameter of the hyperechogenic space around the nerve. This way to measure the optic nerve was taken up in other studies (25, 26).

Ossoinig et al. were the first to suggest to measure the ONSD with ultrasound. They utilized the so called standardized A-scan probe that was performed placing the probe at the temporal limbus over an anesthetized eye (7, 8). This technique utilizes an 8 MHz non-focused probe, with a special S shaped amplification, which showed easily discerned high-reflective spikes at the interface between arachnoid and subarachnoid fluid (7, 8).

This technique was later utilized by Saenz et al. (27), who also utilized the so called “30 degrees test” introduced by KC Ossoinig (7, 8), and concluded that A-scan could assist differentiation of mild papilledema from pseudopapilledema (27).

Unfortunately, in some papers, the utilized technique was not specified (28, 29).

### Correlation and Cut-Off in Intracranial Hypertension

The use of ultrasound to evaluate the optic nerve sheath dilatation is a well-established method that help clinicians in the management of intracranial hypertension when exceeding a certain value. In fact, Karl Ossoinig had already proposed this diagnostic tool in the 1970's for the evaluation of intracranial pressure changes in several pathological conditions (7, 8).

In 1997, Hansen et al. (30) also understood the importance of the optic nerve sheath elasticity that allowed a detectable dilation in response to intracranial hypertension. They investigated the optic nerve sheath response to pressure during CSF absorption using repetitive optic nerve ultrasound B-mode scans on 12 patients.

During the years, many other authors have correlated the ONSD increase with ICP elevation and they have tried to find a cut-off that could be reliable in case of intracranial hypertension related to space-occupying lesions (Table 1).

### Optic Nerve Evaluation in the Emergency Department

Many studies have continued to emphasize the importance of optic nerve ultrasound, especially in Emergency Department (ED), where rapid decision-making can be crucial.

Albert et al. (31) found that an ONSD of more than 5.25 mm and an ONSD/ETD (eyeball transverse diameter) ratio of more than 0.232 on initial CT may identify malignant middle cerebral artery (MCA) stroke patients at high risk of developing malignant MCA syndrome, helping to schedule a decompressive craniectomy. These findings were also supported by the study of Güzeldag et al. (32), who claimed the predictive importance of optic nerve ultrasound in the management and follow-up of MCA infarction.

The possibility of associating changes in ONSD assessable by ultrasound with brain death (BD) was evaluated by Topcuoglu et al. (24) and Toscano et al. (33). In fact, they found that ONSD is significantly greater in subjects with BD. However, quantification of ONSD cannot discriminate BD subjects from comatose ones with raised ICP with a 100% certainty.

In addition, hyperacute stage of intracerebral hemorrhage (34–36), acutely fluctuating intracranial pressure (37), effect of osmotherapy (mannitol administration) (38) and use of Mesalazine (39, 40) are also all factors to concern when measuring the ONSD due to the possibility of rapid change in the obtained values. This demonstrated the optic nerve sheath sensibility to rapid ICP increase or decrease (41).

Kitano et al. (42) analyzed the ONSD enlargement due to vasogenic edema in a case of hypertensive encephalopathy either with no intracranial hypertension, and they demonstrated its lowering after reduction of patient's blood pressure.

### Traumatic Brain Injury

Increased ICP after TBI requires rapid recognition to allow for appropriate treatments. Bedside ultrasound, which is available in most trauma units, should be feasible and quickly performed. For this reason, ultrasound optic nerve examination may provide useful information to guide diagnosis and therapy.

Most of the examined papers concerning brain injury, found a good correlation between ONSD and ICP (43–46).

Good sensitivity and specificity in the detection of increased ICP was found, even if the cut-off values where very different among the papers. Most of the papers agreed in determining the cut-off for the presence of increased ICP when ONSD was more than 5.0 mm (46–49), whereas other suggested different values, such as 4.1 mm (50), 5.205 mm (51), 5.7 mm (52), 5.86 mm (53), 5.95 mm (54), 6.6 mm (55), and 7 mm (56).

Moreover, Cardim et al. (57) found ONSD cut-off values should not be adjusted for sex or age for the ICP assessment in TBI patients.

On the contrary, Strumwasser et al. (58), correlating ONSD and ICP, found a weak connection between ONSD and ICP, suggesting that sonographic ONSD measurements were not reliable as a surrogate for elevated ICP in the absence of invasive monitoring. Similar results were obtained by Martin et al. (59) that, attempting to evaluate the reliability of ultrasound ONSD, and pulsatility index calculation with transcranial Doppler found that both tools did not allow clinicians to rigorously ascertain early ICP increase.

Some others suggested to use ONSD increase together with other parameters to increase the usefulness of non-invasive diagnostic tool.

Singer et al. (60) suggested to combine ONSD measurement with pupillometry and transcranial ultrasonographic Doppler to estimate and follow ICP in patients with severe TBI.

Robba et al. (61) recommended to combine ONSD ultrasonography and transcranial venous straight sinus Doppler to identify critical patients with increased ICP. In another paper, the same authors suggested to utilize the so called “diastolic arterial formula” (62), which include the ONSD measurement, with a sensitivity and specificity of 85%.

The same research group (63) proposed to utilize ICP values, ultrasound ONSD and the pressure reactivity index to obtain information about patients at risk of developing intracranial hypertension and impaired self-regulation.

Du et al. (64) evaluated 52 adults undergoing craniotomy for TBI. The ONSD and ETD of each eyeball were measured by ultrasound and CT within 24 h after an optical probe was placed in the lateral ventricle, finding ONSD/ETD as a good and reliable parameter for predicting increased intracranial pressure in patients with TBI. Concerning ONSD ultrasound measurements, the authors found a mean ONSD of 5.7 mm in TBI patients.

## Discussion

The importance of ocular ultrasound for the assessment, progression, and management of intracranial hypertension in ICUs has been widely discussed (65–67). The ONSD measurement 3 mm behind the eyeball has shown different cut-offs between normality and ICP increase.

This explains why several authors suggest, both in cerebral hypertension due to organic causes and in TBI, to combine ONSD measurements with other parameters, such as ONSDE, ONSDI and ONSD/ETD ratio.

The combination of very different cut-offs and the suggestion to combine ONSD measurements with other parameters, clearly show not only that B-scan technique is not very reliable, but also that ONSD measurements cannot be performed by inexperienced operators, needing a physics and anatomical knowledge of the structures to be examined (68–71).

Among B-scan ultrasonography limitations, there is the presence of the so-called “blooming effect” (72, 73).

It is related to the absence of a standard sensitivity setting, which means that changing the gain during the examination can affect the result. For example, some lesions may appear larger than they really are when the gain is turned down and smaller when the gain is turned up (74).

**Figure 1:**
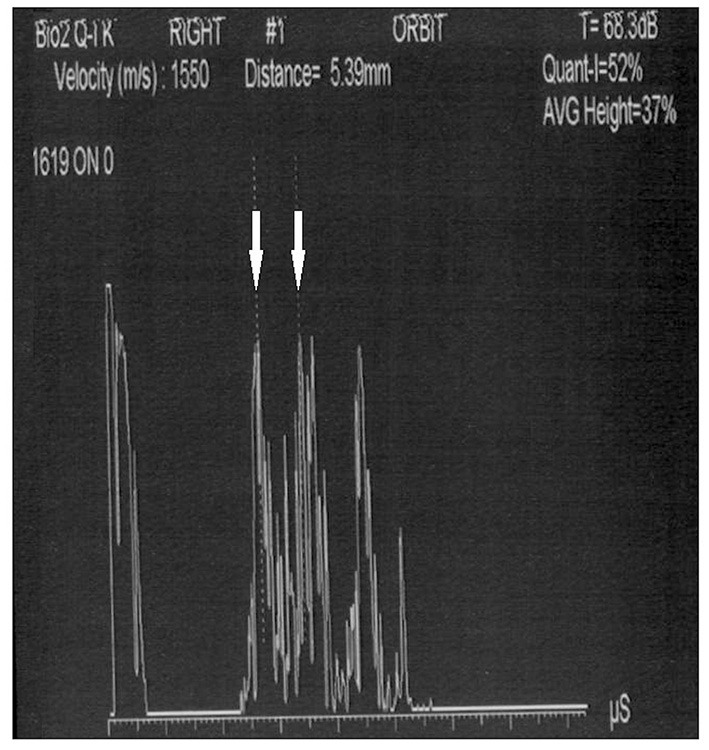
Standardized A-scan image of the optic nerve, showing the two high spikes (white arrows) used to perform ONSD measurement (5.39 mm).

Conversely, A-scan ultrasonography does not have this limitation (75). It is not only suitable for detecting and diagnosing ocular and orbital lesions but also much more reliable in making the measurements. Above all, it is not affected by the blooming effect. This makes it much more suitable for measuring the ONSD because this technique shows easily discernible high reflective spikes from the interface between the arachnoid and subarachnoid fluid (Figure 1). Unfortunately, in almost all the studies considered in this review, B-scan ultrasonography was used, and only Saenz et al. used the standardized A-scan (27), which allows to perform the “30-degree test.”

This dynamic test measures optic nerve thickness in the primary gaze position and after shifting gaze 30 degrees temporally from the primary position (76).

Moreover, this method allows to differentiate whether the enlargement of the optic nerve head is due to intracranial hypertension or to solid lesions or papillitis (77). In fact, in the case of intracranial hypertension, after the “30-degree test,” the stretching of the optic nerve and sheath causes a drastic reduction in the ONSD thickness.

In conclusion, to increase the accuracy and precision, we recommend combining the use of the B-scan method with the standardized A-scan technique. This method requires some training, but is the only way to have precise, repeatable and affordable results.

## Author Contributions

LV, GS, OD'A, and LC analyzed the literature and wrote the original draft. MD, GM, and NR conceived the article and reviewed the manuscript. All authors read and approved the final manuscript.

## Funding

The research was funded with the FARB grant from the University of Salerno.

## Conflict of Interest

The authors declare that the research was conducted in the absence of any commercial or financial relationships that could be construed as a potential conflict of interest.

## Publisher's Note

All claims expressed in this article are solely those of the authors and do not necessarily represent those of their affiliated organizations, or those of the publisher, the editors and the reviewers. Any product that may be evaluated in this article, or claim that may be made by its manufacturer, is not guaranteed or endorsed by the publisher.
